# Metabolic reprogramming of the premalignant colonic mucosa is an early event in carcinogenesis

**DOI:** 10.18632/oncotarget.16129

**Published:** 2017-03-11

**Authors:** Mart Dela Cruz, Sarah Ledbetter, Sanjib Chowdhury, Ashish K. Tiwari, Navneet Momi, Ramesh K. Wali, Charles Bliss, Christopher Huang, David Lichtenstein, Swati Bhattacharya, Anisha Varma-Wilson, Vadim Backman, Hemant K. Roy

**Affiliations:** ^1^ Section of Gastroenterology, Boston University Medical Center, Boston, Massachusetts, USA; ^2^ Department of Biomedical Engineering, Northwestern University, Evanston, Illinois, USA

**Keywords:** colorectal carcinoma, field carcinogenesis, metabolism, metabolic reprogramming, Warburg effect

## Abstract

**Background:**

Colorectal cancer (CRC) is the second leading cause of cancer-related mortality in the United States. There is an increasing need for the identification of biomarkers of pre-malignant and early stage CRC to improve risk-stratification and screening recommendations. In this study, we investigated the possibility of metabolic and mitochondrial reprogramming early in the pre-malignant colorectal field.

**Methods:**

Rectal biopsies were taken from 81 patients undergoing screening colonoscopy, and gene expression of metabolic and mitochondrial markers were assessed using real time quantitative PCR. Validation studies were performed in two different animal models of colon carcinogenesis: Pirc rats and AOM-treated rats.

**Results:**

We found evidence of a Warburg effect in the normal-appearing rectal mucosa of patients harboring precancerous lesions elsewhere in the colon compared to control patients, with a significant increase in *HIF1a*, *SLC2A1 (referred to as GLUT1)*, *PKM2*, and *LDHA*. We also found evidence of early mitochondrial changes in the colorectal field of patients harboring pre-cancerous lesions, with significantly increased mitochondrial gene expression of *DRP1* (fission), *OPA1* (fusion), *PGC1*-a (biogenesis), *UCP2* (uncoupling) and mtND1 (copy number). Similar results were observed in the two different animal models.

**Conclusions:**

These results demonstrate for the first time evidence of early Warburg-like metabolic changes as well as changes in mitochondrial function, dynamics and mtDNA copy number in endoscopically normal premalignant colorectal mucosal field. These findings provide an opportunity for the development of metabolic biomarkers that could be used for improving screening recommendations and risk-stratification. This also provides a potential target for novel chemopreventive strategies in the pre-malignant colorectal field.

## INTRODUCTION

Colorectal carcinoma (CRC) is the second leading cause of cancer-related mortality in the United States [1]. While CRC-related mortality can be greatly reduced when detected at early stages, advanced stages typically have a poor prognosis [2]. Early diagnosis is clearly imperative for the best outcomes. Screening colonoscopy and biopsy remains the gold standard for effective early diagnosis of CRC, however this approach is costly, invasive and underutilized by those at highest risk [3]. There is increasing interest in identifying biomarkers of early stage CRC to improve risk-stratification and screening recommendations.

Our laboratory has focused on identifying early stages of CRC using a field carcinogenesis model. In brief, field carcinogenesis (a.k.a. field of injury, field effect, field defect etc.) posits that the exogenous (diet, smoking, obesity etc.) and endogenous (genetic structure) risk factors lead to a permissive molecular environment (genetic, epigenetic etc.) throughout the colon that allows for focal neoplastic transformation (related to the stochastic event such as mutations occurring in adenomatous polyposis coli gene etc.). This “condemned mucosa” has been corroborated by genomic, proteomic, methylation and micro-nanoarchitectural changes in mucosa that appears normal endoscopically and by conventional light microscopy [3]. It is clinically well established and is the biological basis for surveillance colonoscopy (more frequent colonoscopies for patients with a past lesion that has been removed).

While the molecular aspects of field carcinogenesis are incontrovertible, little is known regarding the physiological correlates. Importantly, while metabolic alterations are a hallmark of frank cancer, the early premalignant changes have been largely unexplored. Our group noted that using a novel biophotonics technology, polarization gated spectroscopy (PGS) that there is an early and diffuse augmentation of the peri-cryptal capillary circulation which we termed early increase in blood supply (EIBS). We noted that EIBS from the rectum was a robust marker for neoplasia throughout the colon and with regards to biological determinants, we have noted induction of *iNOS* and *HIF1*α [4–10].

Since blood flow is intimately coupled to metabolic status, these findings suggest the potential for metabolic dysregulation. Metabolic dysregulation is well established in cancers and has recently emerged as one of the hallmarks of cancer [11–13]. Otto von Warburg first discovered that cancer cells tended to ferment glucose into lactate rather than using the metabolic products of glycolysis for oxidative phosphorylation (OXPHOS), even in the presence of sufficient oxygen [14]. This increase in aerobic glycolysis, known as the Warburg Effect, has been observed in many cancers including CRC [11, 12].

Teleologically, it appears that this loss in ATP generating capability related to Warburg (Kreb's cycle yields much more ATP than glycolysis) is offset by production of multiple anabolic products needed for proliferation. The relevance of metabolic changes in CRC carcinogenesis is supported by studies that show significant changes in multiple metabolic pathways in CRC tissues including nucleotide metabolism, amino acid metabolism, fatty acid metabolism and carbohydrate metabolism [15–17]. Accumulating evidence also suggests that metabolic changes are relevant in colorectal field carcinogenesis. Backshall et al found significant metabolite differences, including an elevation in whole tissue lactate, in morphologically normal/non-tumor tissue from 10 weeks old APC min/+ mice with low tumor burden compared to wild type mice. The authors suggested these metabolic changes could be related the reduction in APC function and likely occurred prior to LOH [18]. This is especially relevant in CRC as APC loss is commonly an early event in the progression of CRC carcinogenesis. Leclerc et al found transcriptomic changes associated with multiple metabolic pathways, including glucose metabolism and lipid metabolism, in the normal intestine of mice with high tumor susceptibility compared to those with low tumor susceptibility [19]. Rao et al recently used a mouse model of CIN (haploinsufficient Sgo1 mice) to show transcriptomic changes in the normal appearing colonic mucosa of mice injected with AOM, including upregulation of pathways involved in lipid metabolism, insulin signaling and PPAR pathways [20].

From a mitochondrial perspective, there is limited data on early changes. In ulcerative colitis, a study suggested that there was a non-monotonic (U shaped) curve between mitochondrial number and future risk of neoplasia elsewhere in the colon [21]. Mitochondrial copy number tends to decrease in frank CRCs.

We, therefore, wanted to investigate the metabolic alterations in early colon carcinogenesis. We took a multi-faceted approach utilizing two well validated animal models, the azoxymethane (AOM) treated rat model and the genetic polyposis in rat colon (PIRC rat, which contains a germline mutation in APC, the initiating event in most CRCs). We complemented this with human rectal biopsy data. In this study, we examined the expression of glycolytic markers (*HIF1α, GLUT1, PKM2* and *LDHA*) as well as markers of mitochondrial dynamics (*DRP1* and *OPA1*), mitochondrial biogenesis (*PGC1*-α), mitochondrial uncoupling (*UCP2*) and mitochondrial DNA (mtDNA) copy number (mtND1). We demonstrate for the first time evidence of early Warburg-like changes in metabolism as well as early alterations in mitochondrial function, dynamics, biogenesis and mtDNA copy number in premalignant colorectal field carcinogenesis.

## RESULTS

### Patient demographics

Rectal biopsies were obtained from 81 patients undergoing colonoscopy. 43 of these patients were found to have no lesions, benign lesions or low risk lesions, and thus categorized as control. 38 of these patients were found to have one or more pre-cancerous lesions: 9 right or transverse hyperplastic polyps, 26 tubular adenomas, and 3 sessile serrated adenomas.

### Metabolic and mitochondrial changes in patient rectal biopsies

#### *HIF1α* expression

**Figure 1:**
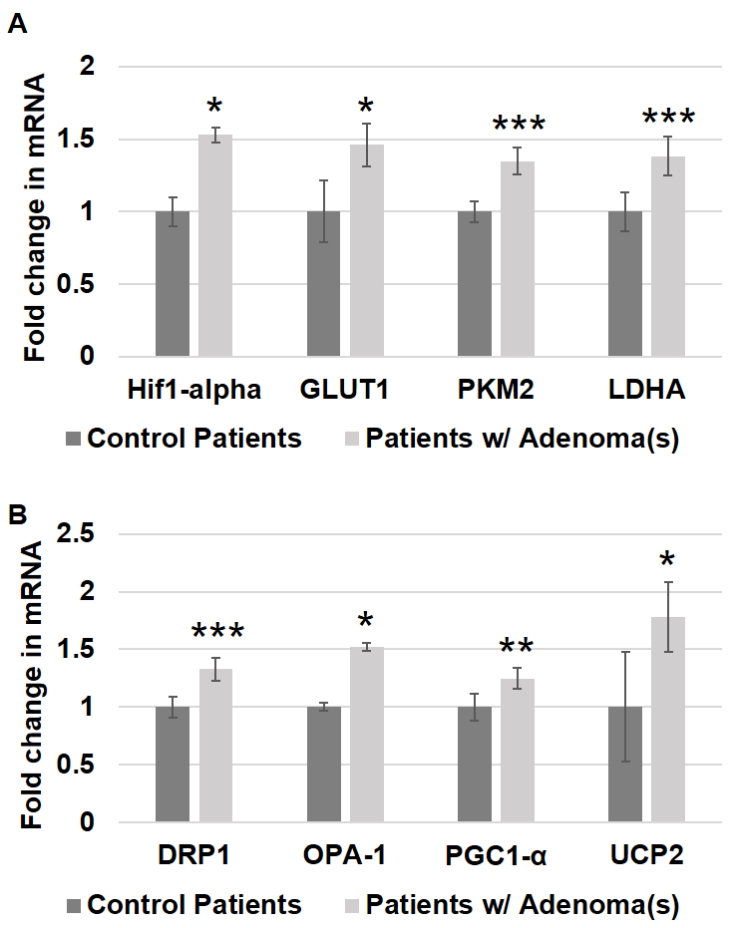
Metabolic and mitochondrial changes in patient rectal biopsies from uninvolved mucosa Messenger RNA (mRNA) expression of **A**. metabolic markers - HIF1α, GLUT-1, PKM2 and LDHA and **B**. mitochondrial markers - DRP1, OPA-1, PGC1-α and UCP2 was measured in rectal uninvolved mucosal biopsies collected from patients with (*n* = 38) or without (*n* = 43) any adenomas. The error bar represents standard error of mean (SEM). Statistical significance is denoted as * = *p* < 0.05; ** = *p* < 0.01; *** = *p* < 0.001.

*HIF1α* is an important transcription factor that is overexpressed in CRC tissues and is known to increase the expression of many factors known to occur in CRC carcinogenesis that contribute to increased aerobic glycolysis as well as angiogenesis [22–25]. *HIF1α* expression induces the expression of multiple genes that increase glucose uptake, increase glycolysis, increase pyruvate fermentation into lactate and decrease pyruvate metabolism by the mitochondria [25]. To investigate whether this increase in *HIF1α* is an early event in CRC field carcinogenesis, we measured the expression of *HIF1α* in patient rectal biopsies. Patients harboring precancerous lesions showed 1.53 fold increase in *HIF1α* expression *p* < 0.05 in their rectal mucosa compared to patients without precancerous lesions (Figure 1A).

#### *GLUT1* expression

*SCL2A1 (referred in the paper as GLUT1*) is a glucose transporter responsible for basal uptake of glucose into cells. Its expression is known to increase in carcinogenesis and hypoxia, both of which are thought to be mediated through *HIF1α* [22]. *GLUT1* has been shown to be overexpressed in CRC tissues, and it is associated with poor prognosis in patients with CRC [26]. This increase in *GLUT1* expression allows cells to increase glucose uptake, which is necessary to supply the glucose needed for the increased glycolysis observed in CRC cells. In order to determine if *GLUT1* increases early in CRC field carcinogenesis, we measured the expression of *GLUT1* in patient rectal biopsies. Patients with precancerous lesions had 1.46 fold increase in *GLUT1* expression *p* < 0.05 in their rectal mucosa compared to patients without precancerous lesions (Figure 1A).

#### *PKM2* expression

Pyruvate Kinase (PK) is a glycolytic enzyme that catalyzes the final step of glycolysis, resulting in the production of ATP and pyruvate. *PKM2* is an isoform of this enzyme that is predominantly expressed in cancer cells, and its expression is induced by *HIF1α* [25]. This isoform is less effective at promoting glycolysis than *PKM1*, and it is thought to promote tumorigenesis by shunting glycolytic precursors into anabolic pathways [11]. *PKM2* levels are elevated in CRC [27], and in patients with CRC, *PKM2* levels are associated with TNM stage and predict poor prognosis [27, 28]. In order to determine if an increase in *PKM2* occurs early in CRC field carcinogenesis, we measured the expression of *PKM2* in patient rectal biopsies. There was a 1.35 fold increase in *PKM2* expression *p* = 0.0004 in the rectal mucosa of patients with precancerous lesions compared to patients without precancerous lesions (Figure 1A).

#### *LDHA* expression

Lactate Dehydrogenase (*LDHA*) is an enzyme that catalyzes pyruvate fermentation into lactate. Importantly, this reaction also generates NAD+ from cytosolic NADH, and adequate levels of NAD+ are necessary for glycolysis. Oxidative phosphorylation, in contrast, uses pyruvate and cytosolic NADH are used for oxygen-dependent ATP in production mitochondria. Increased glycolysis and decreased oxidative phosphorylation in CRC results in increased lactate production. Studies have demonstrated an increased expression of *LDHA* in CRC tissues [29], and *LDHA* expression can be induced by HIF1α [25, 29]. In order to determine if *LDHA* increases early in CRC field carcinogenesis, we measured the expression of *LDHA* in patient rectal biopsies. Patients harboring precancerous lesions had 1.38 fold increased *LDHA* expression *p* = 0.001 in their rectal mucosa compared to patients without precancerous lesions (Figure 1A).

#### Mitochondrial dynamics

Mitochondria exist in dynamic networks that continuously join and divide [30, 31]. Mitochondrial fission results in increased fragmentation of mitochondria. Fission promotes cellular proliferation by ensuring equitable distribution of mitochondria into the daughter cells of actively dividing cells, and it facilitates mitophagy for the elimination of damaged mitochondria [30, 31]. Fission is mediated by *DRP1*, and *DRP1* activation is promoted by *HIF1α* [30, 31]. Mitochondrial fusion creates more interconnected networks of mitochondria, which increases mitochondrial oxidative capacity and dilutes mtDNA mutations [30]. Mitochondrial fusion is mediated by several factors including *OPA1*. In order to determine if changes in mitochondrial dynamics occur early in CRC field carcinogenesis, we measured the expression of *DRP1* and *OPA1* in patient rectal biopsies. Patients with precancerous lesions had 1.33 fold increased *DRP1* expression *p* = 0.005 and a 1.52 fold increased *OPA1* expression *p* < 0.04 in their rectal mucosa compared to patients without precancerous lesions (Figure 1B).

#### Mitochondrial biogenesis

Mitochondrial numbers are regulated to respond to the energy needs of the cell and to compensate for cellular damage [30, 31]. Studies have shown that CRC cells have an increased number of mitochondria [21, 32, 33]. Increased mitochondrial numbers are promoted by *PGC1*-α, the master regulator of mitochondrial biogenesis. In order to investigate mitochondrial number in early premalignant CRC field carcinogenesis, we measured the expression of *PGC1*-α in patient rectal biopsies. Patients harboring precancerous lesions showed 1.25 fold increase in *PGC1*-α expression *p* < 0.006 in their rectal mucosa compared to patients without precancerous lesions (Figure 1B).

#### *UCP2* expression

ATP synthesis in the mitochondria utilizes the electrochemical gradient of protons across the inner mitochondrial membrane to drive ATP synthesis *via* proton flux through the mitochondrial membrane protein ATP Synthase. Uncoupling proteins (UCPs) are mitochondrial membrane proteins that provide an alternative route for protons to flow across the mitochondrial membrane without generating ATP, thereby uncoupling proton flux from ATP synthesis. *UCP2* is an uncoupling protein isoform that has been shown to be overexpressed in adenomas and CRC in a stage-dependent manner [34, 35]. In addition to reducing mitochondrial ATP production through uncoupling, *UCP2* may also reduce OXPHOS by promoting pyruvate efflux from the mitochondria [34]. In order to determine if *UCP2* increases early in CRC field carcinogenesis, we measured the expression of *UCP2* in patient rectal biopsies. *UCP2* expression was 1.78 fold higher *p* < 0.02 in the rectal mucosa of patients harboring precancerous lesions compared to patients without precancerous lesions (Figure 1B).

### Metabolic and mitochondrial changes in animal models of CRC

**Figure 2:**
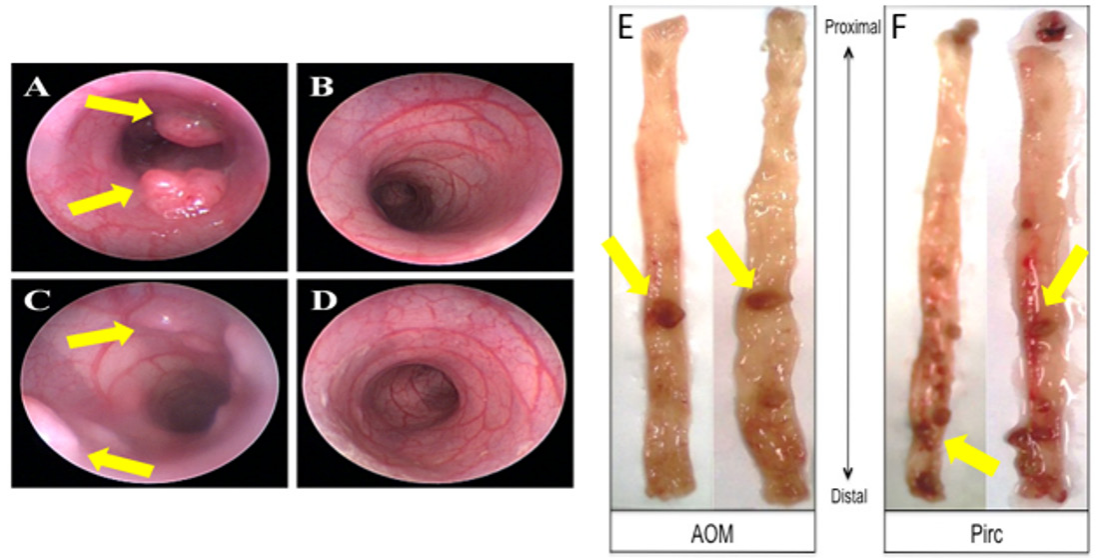
Representative animal model images showing colonoscopy and tumors **A**.-**D**. Colonoscopy images from animals with tumors and control animals; **E**. AOM-induced tumor images and **F**. PIRC rat tumor images in the colon.

**Figure 3:**
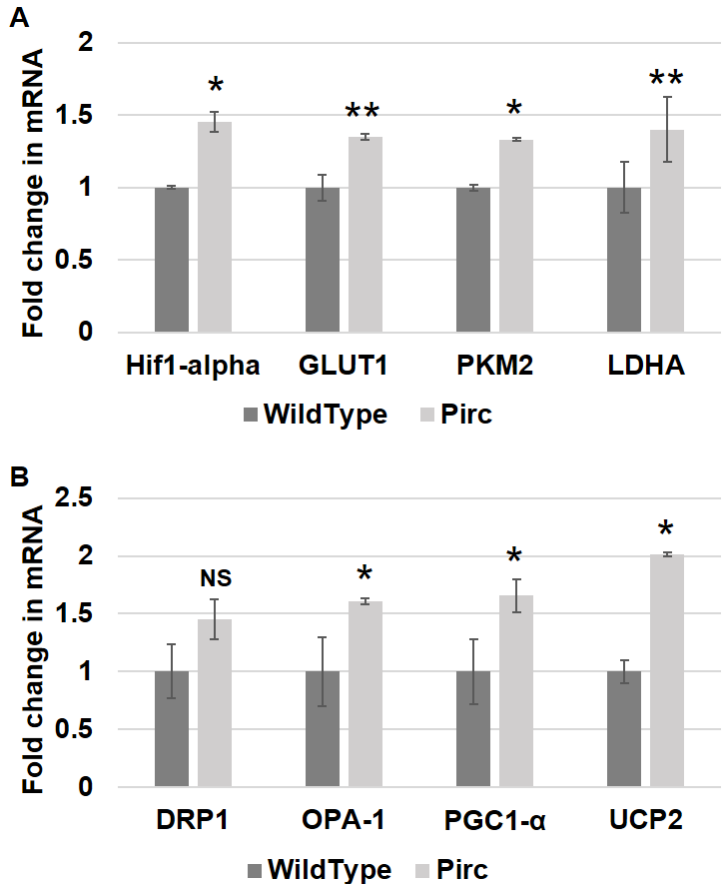
Metabolic and mitochondrial changes in PIRC rat model Messenger RNA (mRNA) expression of **A**. metabolic markers - HIF1α, GLUT-1, PKM2 and LDHA and **B**. mitochondrial markers - DRP1, OPA-1, PGC1-α and UCP2 was measured in rectal uninvolved mucosal biopsies collected from PIRC rats with tumors (*n* = 6) or age-matched control rats (*n* = 6). The error bar represents standard error of mean (SEM). Statistical significance is denoted as * = *p* < 0.05; ** = *p* < 0.01; *** = *p* < 0.001.

We further investigated whether metabolic and mitochondrial changes occurred in two animal models of CRC. Polyposis in Rat Colon (Pirc) Rats, which have a genetic mutation in *APC*, were used as a model for sporadic CRC since the majority of sporadic CRCs are attributed to *APC* truncation. Pirc rat tumor images are shown in Figure 2. Pirc rats harboring adenomas/tumors had a significant increase in glycolytic markers in their noninvolved colorectal mucosa compared to Pirc rats without adenomas/tumors, including *HIF1α* (1.45 fold, *p* = 0.018), *GLUT1* (1.35 fold, *p* = 0.003), *PKM2* (1.33 fold, *p* = 0.05) and *LDHA* (1.4 fold, *p* = 0.003) (Figure 3A). In terms of mitochondrial changes, Pirc rats harboring adenomas/tumors had increased mitochondrial fission (1.45 fold increase in *DRP1*, *p* = 0.09), increased mitochondrial fusion (1.61 fold decrease in *OPA1*, *p* = 0.022), increased mitochondrial biogenesis (1.65 fold increase in *PGC1*-α, *p* = 0.038) and mitochondrial uncoupling (2.01 fold increase in *UCP2*, *p* = 0.05) (Figure 3B).

**Figure 4:**
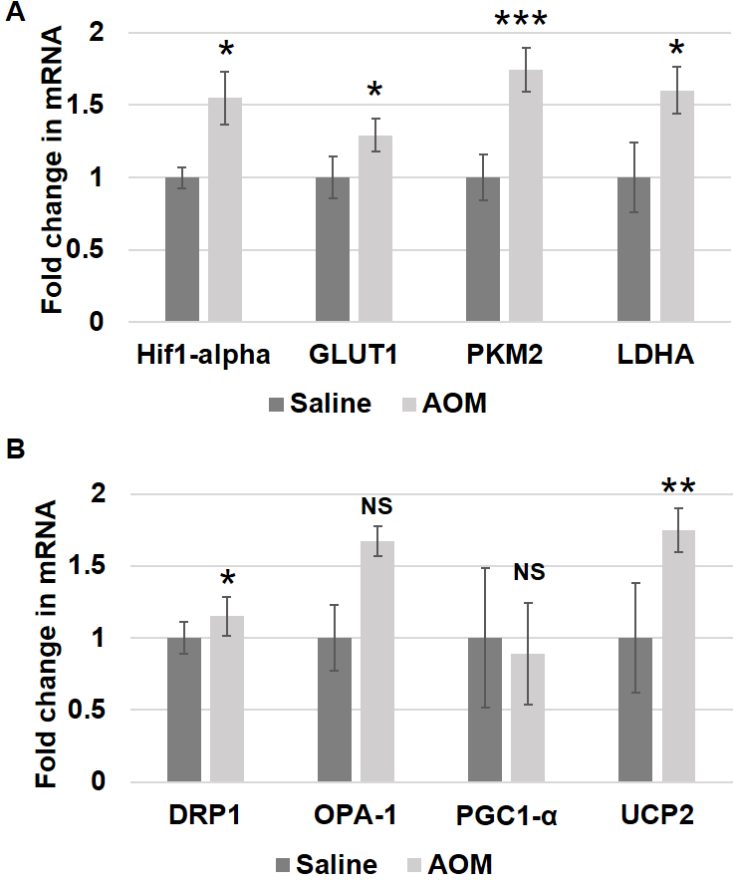
Metabolic and mitochondrial changes in AOM rat models Messenger RNA (mRNA) expression of **A**. metabolic markers - HIF1α, GLUT-1, PKM2 and LDHA and **B**. mitochondrial markers - DRP1, OPA-1, PGC1-α and UCP2 was measured in rectal uninvolved mucosal biopsies collected from AOM-treated rats (*n* = 6) or control saline-treated rats (*n* = 6). The error bar represents standard error of mean (SEM). Statistical significance is denoted as * = *p* < 0.05; ** = *p* < 0.01; *** = *p* < 0.001.

**Figure 5:**
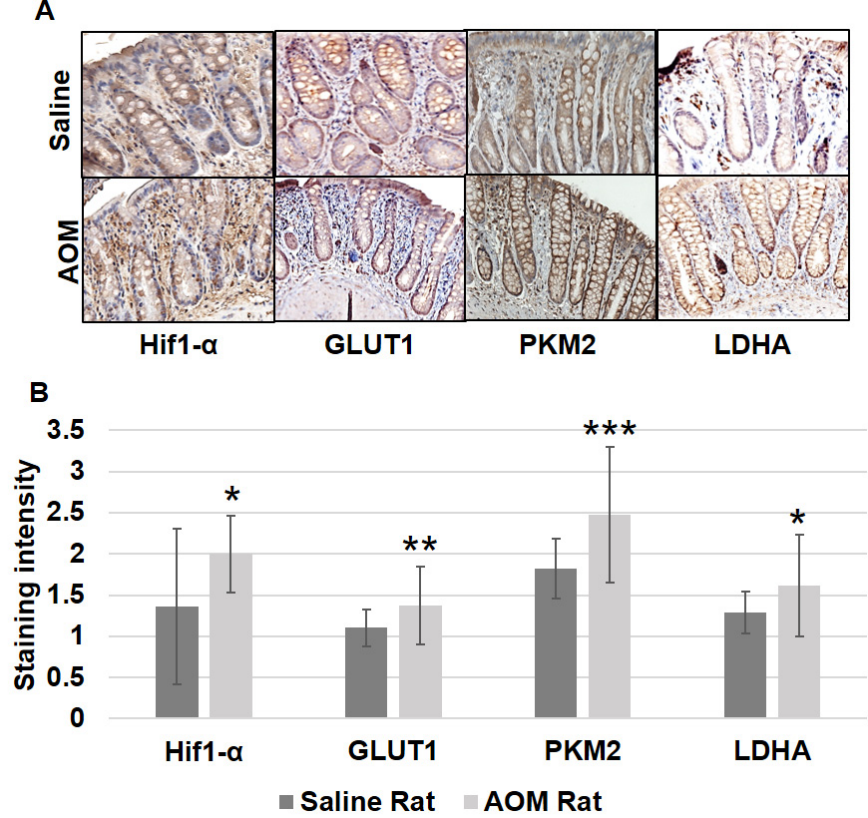
Metabolic changes in uninvolved mucosa by IHC in AOM model Expression of HIF1α, GLUT-1, PKM2 and LDHA by immunohistochemical staining was performed as described in Materials and Methods Section. Ten optical fields from each specimen were scored (0-3; with 0 being no intensity and 3 very strong). The error bar represents standard error of mean (SEM). Statistical significance is denoted as * = *p* < 0.05; ** = *p* < 0.01; *** = *p* < 0.001.

We further explored these metabolic and mitochondrial changes in the premalignant colorectal field in an AOM-induced CRC model. AOM-treated rat tumor images are shown in Figure 2. Rats injected with AOM twice weekly had a significant increase in glycolytic markers in their noninvolved colorectal mucosa compared to rats injected with saline, including HIF1α (1.55 fold, *p* = 0.04), *GLUT1* (1.29 fold, *p* = 0.05), *PKM2* (1.74 fold, *p* = 0.001) and *LDHA* (1.60 fold, *p* = 0.052) (Figure 4A). In terms of mitochondrial changes, rats injected with AOM had increased mitochondrial fission (1.15 fold increase in *DRP1*, *p* < 0.05), increased mitochondrial fusion (1.67 fold increase in OPA1, *p* = 0.07), decreased mitochondrial biogenesis (0.89 fold increase in *PGC1*-α, *p* = 0.07) and increased mitochondrial uncoupling (1.75 fold increase in *UCP2*, *p* < 0.01) (Figure 4B). We next performed IHC on the metabolic markers to validate the mRNA findings. Rats injected with AOM twice weekly had a significant increase in glycolytic markers in their noninvolved colorectal mucosa compared to rats injected with saline, including HIF1α (1.47 fold, *p* = 0.015), GLUT1 (1.25 fold, *p* = 0.008), PKM2 (1.358 fold, *p* = 0.0003) and LDHA (1.25 fold, *p* = 0.016) (Figure 5A, 5B).

### Mitochondrial DNA copy number in patient samples, PIRC and AOM models

**Figure 6:**
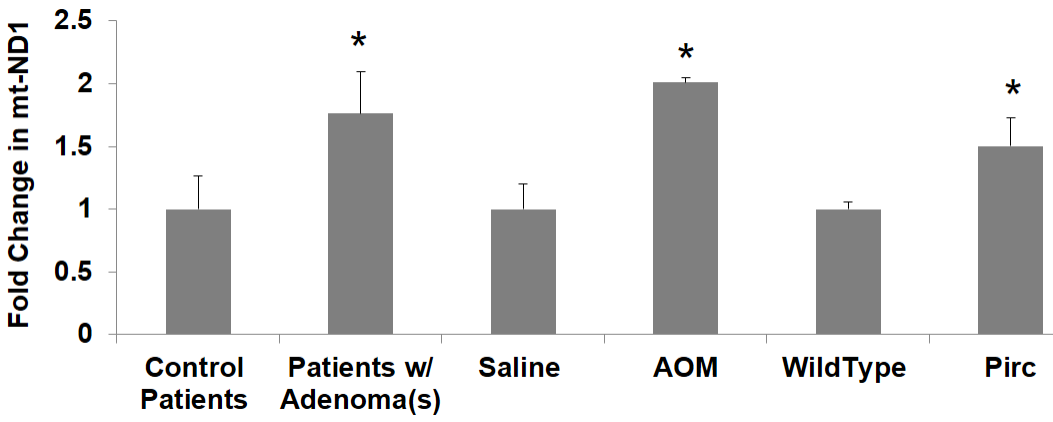
Changes in mitochondrial copy number mt-ND1 in premalignant field in patients and animal models Messenger RNA (mRNA) expression of mitochondrial gene mtND1 was measured in patient rectal biopsies, PIRC and AOM models. The error bar represents standard error of mean (SEM). Statistical significance is denoted as * = *p* < 0.05; ** = *p* < 0.01; *** = *p* < 0.001.

Mitochondria contain multiple copies of mtDNA (2-10 each), and increasing the mtDNA copy number can help compensate for the presence of mtDNA mutations. Studies have shown that CRC tumors have increased mtDNA copy number, and epidemiological studies have also demonstrated a significant association between mtDNA copy number and CRC risk [21, 36]. In order to investigate changes in mitochondrial DNA copy number in early premalignant CRC field carcinogenesis, we quantified expression of the mitochondrial gene mtND1 and normalized this to the expression of the nuclear gene 18S in patient rectal biopsies. Patients harboring precancerous lesions showed 1.76 fold increase in mtND1 expression *p* < 0.05 in their rectal mucosa compared to patients without precancerous lesions (Figure 6). Pirc rats harboring adenomas/tumors had increased mtDNA copy number (1.50 fold increase in mtND1, *p* < 0.05). Rats injected with AOM had increased mtDNA copy number (2.01 fold increase in mtND1, *p* < 0.02) (Figure 6).

The comparative table of percentage change in various metabolic and mitochondrial alterations in human and mouse tissues (AOM and PIRC rat models) have been shown in Table 1 for comprehensive view.

**Table 1:** Comparative table of percentage change in various **A**) metabolic and **B**) mitochondrial alterations in human and mouse tissues

A.				
	Hif1-alpha	GLUT1	PKM2	LDHA
Patients w/ Adenoma(s) vs Control Patients	53% increase	∼46% increase	∼35% increase	38% increase
AOM Rat vs Saline Rat	∼55% increase	29% increase	74% increase	60% increase
Pirc Rat vs Wildtype Rat	50% increase	35% increase	33% increase	40% increase

B.					
	mtND1	DRP1	OPA-1	PGC1-α	UCP2
Patients w/ Adenoma(s) vs Control Patients	76% increase	∼33% increase	52% increase	∼26% increase	78% increase
AOM Rat vs Saline Rat	100% increase	15% increase	∼67% increase	11% decrease	75% increase
Pirc Rat vs Wildtype Rat	50% increase	45% increase	61% increase	∼66% increase	100% increase

## DISCUSSION

Metabolic and mitochondrial reprogramming are known to play an important role in CRC. However, such changes in early premalignant field carcinogenesis have been largely unexplored. We report for the first time evidence of early glycolytic changes as well as early alterations in mitochondrial function, dynamics, biogenesis and mtDNA copy number in premalignant colorectal mucosal field. These physiological changes may propagate mutations as evidence by the role of glucose uptake in K-ras mutations [37].

The changes in glycolytic markers that we observed in the early premalignant colorectal mucosal field are consistent with changes that have been described in CRC, and these changes would be expected to promote increased glycolysis and shunting of glycolytic intermediates into anabolic pathways [11, 22, 25, 26, 29]. Consistent with an early Warburg Effect, our findings suggest an early increase in glucose uptake (*GLUT1*) and pyruvate fermentation into lactate (*LDHA*). We also found an increase in the glycolytic enzyme *PKM2*, an isoform of PK that is predominantly expressed in CRC cells and while slows down glycolysis but fosters the anabolic pathways and thus serves as a hallmark of Warburg effect [11, 27]. By slowing down the final step of glycolysis, *PKM2* may promote carcinogenesis by driving glycolytic intermediates toward anabolic pathways [11].

While the molecular drivers of these early alterations, one logical candidate is the *HIF1α*. This transcription factor that is overexpressed in CRC [23, 24] and is known to induce the expression of multiple glycolytic genes including *GLUT1, PKM2, LDHA* [25]. Low oxygen and high mitochondrial ROS promote increased levels of *HIF1α* [38]. In addition to its role in increased glycolysis, *HIF1α* is also known to stimulate angiogenesis by increasing the expression of proangiogenic factors, which is consistent with our prior data showing EIBS and angiogenesis in premalignant CRC field carcinogenesis [3, 6–8, 39]. Together, these data suggest that an early increase in *HIF1α* expression in the colorectal field induces increased aerobic glycolysis and angiogenesis, which then provides a fertile field for the development of colorectal carcinogenesis.

In addition to evidence of increased glycolysis in the premalignant colorectal field, we also found multiple gene expression changes in the mitochondria responsible for increased uncoupling, increased fission and fusion, increased biogenesis and increased mtDNA copy number. While an increase in mitochondria was not *a priori* what we might have predicted, the data from ulcerative colitis shows a U shaped curve suggests the relationship is quite complex. However, several lines of evidence below suggest mitochondrial function may be compromised [40].

Uncoupling proteins (UCPs) are mitochondrial membrane proteins that allow protons to bypass ATP Synthase and flow across the membrane without generating ATP. *UCP2* is an uncoupling protein isoform that has been shown to be overexpressed in adenomas and CRC in a stage-dependent manner [34, 35]. Our data show that this increase in *UCP2* actually occurs early on in the normal appearing mucosa of the premalignant colorectal field. The role of *UCP2* in carcinogenesis is not well understood [34, 35, 41]. One potential role may be through direct effects on glycolytic metabolites. *UCP2* may promote pyruvate efflux from the mitochondria, thereby restricting its use in OXPHOS [34]. Another potential role is modulation of mitochondrial ROS. *UCP2* is activated by increases in mitochondrial ROS, and it has the effect of reducing of mitochondrial ROS [34, 35]. While mitochondrial ROS is an important signaling molecule that can stimulate proliferation and contribute to tumorigenesis, it can also be toxic to cells and induce apoptosis [31, 34, 38, 40]. CRC cells are known to have increased levels of mitochondrial ROS, and *UCP2* may function to help cancer cells avoid ROS-mediated apoptosis [31, 34, 35, 38, 40].

Mitochondria exist in dynamic networks that continuously join and divide *via* fusion and fission, respectively. These dynamics influence important cellular processes including ATP generation, ROS production, apoptosis, mitophagy, and O2 sensing [30, 31]. The role of mitochondrial dynamics in human disease, including cancer, has recently been reviewed [30, 31]. Mitochondrial fission, which is mediated by *DRP1*, results in fragmentation of mitochondria. Fission facilitates mitophagy for the isolation and elimination of damaged mitochondrial. It is also closely coordinated with mitosis to ensure equal distribution of mitochondria into the daughter cells of actively dividing cells, and thus accelerates cell proliferation. DRP1 activation is promoted by *HIF1α* [30, 31]. Mitochondrial fusion, which is mediated by several factors including mitofusin-1, mitofusin-2 and optic atrophy 1 (*OPA1*), results in more interconnected networks of mitochondrial. This allows for sharing of matrix content, dilution of mtDNA mutations and results in increased mitochondrial oxidative capacity [30]. Our findings of an increased expression of *DRP1* suggest an increase in mitochondrial fission, and this increase in *DRP1* may have been promoted by the increase in *HIF1α*. This increase in fission in the pre-malignant colorectal field may promote increased cellular proliferation and thus would facilitate carcinogenesis. This data is in agreement with other studies that have reported increased fission in cancer cells [30, 31]. In fact, inhibition of fission was shown to decrease proliferation and increase apoptosis in cancer cells and results in lung tumor regression [30].

Mitochondrial numbers are regulated to respond to the energy needs of the cell and to compensate for cellular damage [30, 31]. Studies have shown that CRC cells have an increased number of mitochondria [21, 32, 33]. Increased mitochondrial numbers are promoted by *PGC1*-α, the master regulator of mitochondrial biogenesis. We observed an increase in *PGC1*-α expression in the normal appearing colorectal mucosa of patients harboring pre-cancerous lesions, which suggests an increase in mitochondrial number. This is consistent with a recent study from Payne et al, which found increased mitochondrial mass in non-neoplastic colonic mucosal field of patients with CRC. Authors propose that the increase in mitochondrial mass may have been driven by oxidative stress during the early stages of carcinogenesis since oxidative stress is known to increase mitochondrial mass [33]. In a study on patients with ulcerative colitis, Ussakli et al observed that *PGC1*-α, mtDNA copy number, cytochrome c oxidase and several mitochondrial proteins were significantly increased in CRC tissue compared to low or high grade dysplasia. However, in the nondysplastic mucosa of patients with dysplasia, they observed a progressive reduction in these same markers with decreasing distance from dysplastic lesion. These authors proposed that there is a preneoplastic reduction in mitochondrial mass, which is later restored in cancer, and is driven by *PGC1*-α [21]. This is in contrast to our data which shows an early increase in the expression of *PGC1*-α and mtDNA copy number in the normal appearing colorectal mucosal field at the pre-cancerous stage of carcinogenesis, suggesting an early increase in mitochondrial biogenesis and mitochondrial number. This discrepancy may be due to the fact that the study population had UC, which may result in a different pattern of mitochondrial changes in pre-malignancy.

Mitochondria contain multiple copies of mtDNA (2-10 each). Studies have shown that CRC tumors have increased mtDNA copy number and epidemiological studies have also demonstrated a positive association between mtDNA copy number and CRC risk [21, 36]. We found evidence of an increased mtDNA copy number in the precancerous colorectal mucosal field compared to controls. Some studies have suggested that the relationship between mtDNA copy number and CRC may be non-linear [21, 36]. An epidemiological study by Thyagarajan et al investigated the relationship of mtDNA copy number in peripheral blood (which correlates to mtDNA copy number in the normal colonic mucosa) and risk of CRC. They demonstrated a U-shaped association between relative mtDNA copy number and CRC risk. Specifically, those with the lowest relative mtDNA copy number and those with the highest relative mtDNA copy number in their peripheral blood had significantly increased risk of CRC [36]. Ussakli et al [21] observed a progressive reduction in mtDNA copy number in the nondysplastic mucosa with decreasing distance from the dysplastic lesion in ulcerative colitis patients with dysplasia. They also found a significant increase in the mtDNA copy number in CRC. These authors proposed that there is a preneoplastic reduction in mitochondrial mass, which is later restored in cancer [21]. The underlying mechanism for this relationship between mtDNA copy number and CRC is not well understood. Increased mtDNA copy number may help mitochondria compensate for increases in oxidative stress and for accumulation of mtDNA mutations, thereby providing a survival benefit in carcinogenesis [36].

Further studies are needed to elucidate the specific mechanisms underlying these metabolic and mitochondrial changes. Our data suggest that an early increase in HIF1α expression in the colorectal field induces increased aerobic glycolysis in the pre-malignant colorectal field. Increased *HIF1α* may also induce an increase in mitochondrial fission *via* promotion of *DRP1*, resulting in lower OXPHOS capacity. This underlying cause of this early increase in *HIF1α* may be related to increased oxidative stress in the colorectal field since increased mitochondrial ROS is known to increase *HIF1α* levels [38]. Increased mitochondrial ROS likely induces an increase in *UCP2*, which may function to prevent excess accumulation of ROS, promote retrograde mitochondrial signaling and/or reduce ATP generation *via* OXPHOS. Oxidative stress is also known to increase mitochondrial mass, which may be the reason we observe an increase in *PGC1*-α. These metabolic and signaling pathways are complex, however, and the underlying mechanism needs to be clarified in future studies. It is important to note that these changes in metabolism and mitochondria occur early in CRC carcinogenesis and precede the development of pre-cancerous lesions. This suggests that they provide a microenvironment in the colorectal field that promotes carcinogenesis.

There are many strengths in this study. The combination of a genetic and carcinogen treated well validated models targeting the premalignant mucosa. This was coupled with pilot human data. The concordance of these discrete lines of evidence is quite powerful. The addition of human samples (rectal biopsies) from a well-annotated cohort highlights the relevance to human colonic neoplastic transformation. The exploration of Warburg by evaluating 4 distinct markers of glycolysis and 3 distinct markers of mitochondria adds to the robustness of these novel findings.

There are some limitations in our study that should be acknowledged. First, we only quantified mRNA levels using RT-PCR except the IHC of metabolic markers for AOM model. While this measure does quantify changes in gene expression, there can clearly be some discordance between message and protein. Somewhat mitigating that is that the protein from the AOM-treated rat nicely recapitulated the mRNA data from humans and Pirc rat. Second, we assessed Warburg through expression of protein markers and not directly by measuring colonic metabolism. That being said, we do have data that in cell culture, recapitulating tumor suppressor gene loss in early colon carcinogenesis was accompanied by Warburg like physiology as demonstrated by Seahorse assays and metabolomics (reference [42] and unpublished data). Third, mitochondrial function, dynamics and mass were studied indirectly *via* alterations in the gene expression of certain markers. Since we did not directly visualize mitochondria or study mitochondrial function directly, it is unknown if changes in these markers reflected observable changes in mitochondrial function, dynamics or mass. Finally, adenoma studies are fraught with complications in that only a small percentage of adenomas would ever go on to CRC. [15]. In addition, given polyp miss rate is well established, some of our controls may actually harbor adenomas. This would, if anything, dampen the effect we are seeing so the fact that most parameters achieved statistical significance is reassuring. It would have also been interesting to see if there was a gradient of metabolic abnormalities with severity of neoplasia but we were underpowered and lacked any patients with CRC as would have been expected in a screening/surveillance cohort.

**Figure 7:**
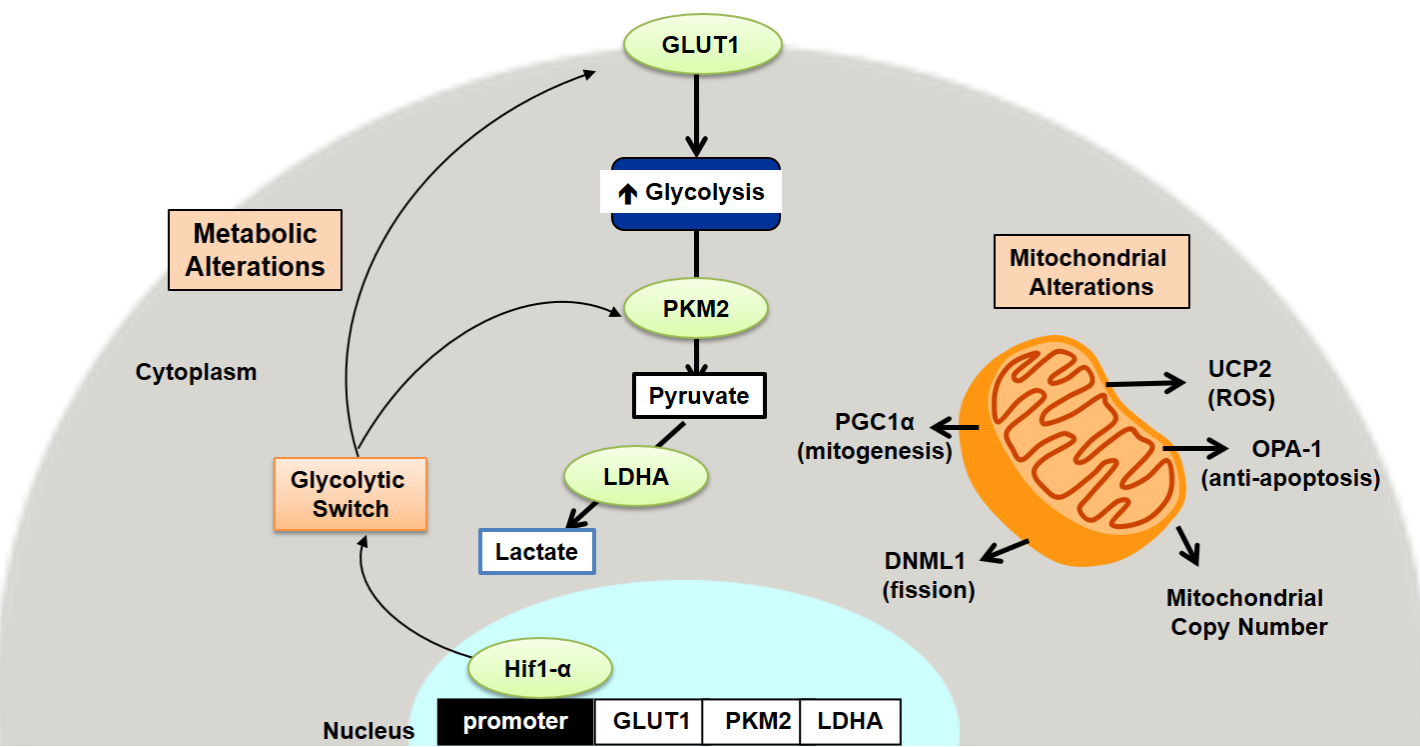
Schematic representation of the metabolic and mitochondrial alterations in early colon carcinogenesis

In conclusion, we demonstrate for the first time evidence that normal appearing premalignant colorectal mucosal field exhibits early Warburg-like metabolic changes as well as changes in mitochondrial function, dynamics and mass (schematic representation shown in Figure 7). These changes may provide a favorable microenvironment in the colorectal field for carcinogenesis to take place. The fact that these changes are observed so early in the pre-malignant colorectal field suggests that it could be a driving process in CRC carcinogenesis. These observations provide an opportunity for the development of metabolic biomarkers that could be used to improve risk-stratification and screening recommendations. This also provides a potential target for novel chemopreventive strategies in the pre-malignant colorectal field.

## MATERIALS AND METHODS

### Human studies

Approval was obtained from the Institutional Review Board at Boston Medical Center and informed consent was obtained from each patient. Patients undergoing routine colonoscopy for screening or surveillance were included and were de-identified in the study. Rectal mucosal biopsies were obtained at the time of screening colonoscopy. Samples were collected in PBS and frozen for preservation and later analysis. Biopsy results of any lesions removed from the colon were recorded. Patients were classified as harboring a precancerous lesion if they had one or more of the following high risk lesions: hyperplastic polyps found in the right or transverse portions of the colon and any type of adenoma). Patients were classified as control if no lesions were found or if all the lesions were found to be low risk (benign lesions and left sided hyperplastic polyps). Patients with incomplete colonoscopies were excluded from analysis.

### Animal studies

Animal protocols were reviewed and approved by the Institutional Animal Care and Use Committee (IACUC) of NorthShore University HealthSystem (Evanston, IL). Animal housing was climate-controlled (ambient temperature of 25 degrees C, humidity of 60%) and at had a 12-hour light/dark cycle.

### Azoxymethane (AOM) rat model

The AOM rat is a well validated chemically induced model of colon carcinogenesis. Germane aberrations caused by AOM include K-ras mutation, leading to alterations downstream to PI3K/AKT and MAPK pathways. Beta catenin is also mutation, ablating the ability of GSK to degrade beta catenin [43]. Although APC is not mutation in this model (where ∼75-80% of sporadic CRC possess), it is still consistent with Vogelstein's model of CRC (k-ras mutations, beta catenin perturbations) [44]. Phenotypically, the AOM model recapitulates human CRC carcinogenesis in ACF formation, distal tumor predilection, as well as histological features of adenomas formed in this rodent model [45]. Twelve male rats of Fisher F-344 background were procured from Harlan Teklad (Madison, Wisconsin) and maintained on a diet of AIN76-A rodent chow (Harlan Teklad) with ad libitum access to water. These rats were randomized to either 2 weekly intraperitoneal injections of azoxymethane (AOM, administered at a concentration of 15mg/kg of body weight) or saline. Serial colonoscopies were performed on the rodents for adenoma detection/frequency. Rats were housed for 40 weeks and euthanized. Colons were excised and cleansed with ice cold phosphate saline buffer (PBS). Colonic epithelial mucosa was collected for genetic analysis.

### Polyposis in rat colon (Pirc) rat model

The polyposis in rat colon (Pirc) rat is a genetic model of Familial Adenomatous Polyposis (FAP), derived from *N*-ethyl-*N*-nitrosourea (ENU) mutated Fischer 344 rats to develop a mutation in APC^Δ1137^, causing a truncated at the third amino acid of the second 15-aa β-catenin binding domain (a region highly conserved in vertebrates) [46]. Phenotypically, tumors from these animals closely resemble the human disease in local invasion, tumor multiplicity as well as anatomical and histological features. We chose a ∼8 mo (32 wk) timepoint in this study from colonoscopic findings (tumor frequency) to compare our commercially obtained rodents from the original lab reports (similar tumor frequency). Furthermore, it has been previously reported that tumors from this timepoint would exhibit high grade dysplasia with local invasion^4^. Lastly these rodents become moribund from disease (bowel obstruction, etc) allowing for longitudinal studies for colon carcinogenesis. Although this is a polyposis model, 75-80% of sporadic CRC cases are thought to arise from APC truncation/LOH. Although FAP accounts for ∼1% of all CRC cases, there is a 100% penetrance in these patients. Six Pirc Rats were obtained from Taconic Laboratories (Hudson, NY) at an age of 10 weeks. These rodents have a genetic mutation at the APC codon 1137, resulting in the development of multiple colonic neoplasms at 3-4 months of age. This is models the human disease FAP but is also a useful model for CRC as ∼80% of sporadic CRCs are attributed to APC truncation. Six age-matched controls (Fisher F-344 rats consistent with Pirc rat background) were also obtained (Taconic Laboratories, Hudson, NY). Serial colonoscopies were performed on the rodents for adenoma detection. These animals were euthanized at 24 weeks. Colons were excised and flushed with PBS. Colonic epithelial mucosa was obtained for analysis.

### RNA and DNA isolation

All colorectal mucosal samples (animal and human) were homogenized by mechanical disruption and stored in Trizol for RNA stabilization. RNA and DNA were isolated with Ribopure RNA isolation Kit and quantified with NanoDrop (Fisher Scientific, Hanover Park, IL) as per manufacturer's protocol.

### Gene expression

cDNA was synthesized using the High Capacity cDNA Synthesis Kit (Life Tech, Foster City, CA) and Step-One Plus Thermocycler (Life Tech, Foster City, CA). Gene expression was quantified by Real Time PCR using gene specific Taqman primers and Taqman Universal Master Mix. The comparative (2^−ΔΔCt^) method was used for quantification with beta actin expression as a standard (RQ Manager 1.2.1, Life Tech, Foster City, CA).

### Immunohistochemistry (IHC)

To determine the expression of metabolic markers HIF1α, GLUT1, PKM2 and LDHA, IHC was performed on formalin fixed rectal biopsies of AOM-treated and control rats that were embedded in paraffin blocks, sectioned (4μm thick) and mounted on Superfrost^+^ glass slides. The slides were deparaffinized by heating at 60°C (∼1 hour) and two washes in xylene followed by rehydrating with graded alcohol washes. The tissue sections were subjected to antigen-epitope retrieval by pressure microwaving (Nordic-Ware) at high power setting (2 × 9 minutes) in antigen unmasking solution (Vector Laboratories, Burlingame, CA). After quenching the endogenous peroxidase activity in 3% hydrogen peroxide, the slides were incubated in 5% horse serum for 2-3 hours at room temperature to block non-specific binding. The sections were then incubated with anti- HIF1α, -GLUT1, -PKM2 and -LDHA antibodies overnight at 4°C. After standard PBS washings, the sections were incubated with the universal biotinylated secondary antibody (1:2000) for 30min followed by avidin-biotin peroxidase using Vectastatin Elite ABC Reagent Kit (Vector Laboratories, CA). Finally, the antigen-specific brown staining was developed by exposing the sections to 3, 3′-diaminobenzidine (DAB) as the chromogen substrate (for 1-3 minutes). For the negative controls, duplicate sections on the same slide were processed in the absence of the primary antibody. The color intensity was scored on a scale of 0-3 by an investigator blinded to colonoscopic findings for rectal biopsies of AOM-treated and control rats.

### Reagents

Primary antibodies for immunohistochemistry were obtained from ABCAM (Cambridge, MA). Immunohistochemistry was performed using Vectastain Elite ABC HRP Kit and visualized with 3,3′-diaminobenzidine. Quantitative real time PCR were performed using Taman Gene Expression Assays (Life Tech, Foster City, CA).

### Statistical analysis

Differences in gene expression were analyzed using two-tailed Student's *t*-test.

## Abbreviations

CRC: Colorectal carcinoma
PGS: Polarization gated spectroscopy
EIBS: Early increase in blood supply
OXPHOS: Oxidative phosphorylation
AOM: Azoxymethane
PIRC: Polyposis in rat colon
HIF1α: Hypoxia Inducible factor 1-alpha
GLUT1: Glucose transporter 1
PK: Pyruvate Kinase
LDHA: Lactate Dehydrogenase
DRP1: Dynamic-related protein 1
OPA1: Optic atrophy 1
PGC1-α: Peroxisome proliferator-activated receptor c coactivator 1-alpha
UCP2: Uncoupling protein 2
